# The Eight-Hours' Day in Private Nursing

**Published:** 1919-09-27

**Authors:** 


### THE MATRONS' AND SISTERS' DEPARTMENT.

### THE EIGHT-HOURS' DAY IN PRIVATE NURSING.

IT is generally recognised that the eight-hours' day cannot be restricted merely to nurses in training. The medical officer of one of the largest nurses' co-operations in London has recently testified to the evil effect on the well-being of the nurses under his supervision of the long hours to which they are exposed. He described them as filled with a painful lassitude between their cases; as prematurely aged and completely lacking in *joie de vivre* after a few years' experience of the "best" medical and surgical nursing. No one will be prepared to dispute that this is thoroughly bad. Few, we believe, will be prepared to dispute the facts. To find the remedy is likely to prove vastly more difficult than to better the lot of probationers and nurses in hospitals.

An acute case of illness in a private house where supernumerary help is not available demands that two nurses must put in twelve hours each in the twenty-four; ought this to be regarded a normal day's work for the nurse? No doubt there are cases even of acute illness in which a double shift of nurses suffices. Often patients are sufficiently composed and self-reliant to be left during the nurse's meal-times and are considerate enough to allow her in other respects periods of quiescence which are practically rest; often, too, there is a relative or upper servant competent to take over the patient in order to relieve the nurse occasionally during her twelve hours' vigil. But we are to consider the not infrequent cases in which the patient cannot safely be left and demands incessant services, cases in which there is neither relative nor maid who can assist. It is twelve hours' work in such cases which wear down the vitality of the very strongest women. It is these cases which imperatively call for the introduction of a three-shift system. They are commoner than is usually supposed. Nursing institutes in London minister largely to the hotel or apartment house population. Here no extraneous help is available. Moreover, the nurse's help is invoked continually in sudden seizures or in the last stages of incurable disease, in order that she may ease the last days or hours when relatives are foredone. Here no limit is placed on the endurance of the fresh comer. The mental and physical strain on women who pass from one such brief case to another, frequently without pause or relief, is greater than any should be called on to endure. We call to mind the spirit of strained endurance visible in the eyes of a private nurse who mentioned as a point of interest that she had assisted at three deathbeds in the course of the past week. She was momently expecting a further call. In no case had her hours on duty been limited even to twelve hours. This state of things imperatively calls for reform. Three nurses are not too many for such emergencies. Often one only is supplied.

There is another class of case, that of the general paralytic, the senile or mental case, in which a double shift of nurses is plainly insufficient. Very seldom can any unskilled help be utilised in such maladies. Meals have often to be taken hurriedly, either in or within sound of the sick-room. To demand twelve hours' attendance a day for months at a time from the nurses in charge of such a case is inhuman. It defeats its own aims, for the patient loses the incalculable blessings which flow from the ministrations of a calm, alert individual able to reflect upon his consciousness the inner strength and consolation for which subconsciously he longs. By degrees, instead of a nurse, he finds himself waited upon by a neurotic whose nerve signs are patent to any trained observer, but whose habitual self-control keeps her going. When the nurse is too worn out physically and mentally to control the patient's mind, it is this sick mind which will dominate hers.

But the expense. For such cases as we have described the nurse commands a fee of three or four guineas a week, in addition to full board, lodging, attendance, and allowances, which may be reckoned at another two guineas a week. Three nurses on such terms would cost not far short of £1,000 a year. It is not too much to pay for the entire dedication to one's service of three highly skilled and conscientious women. It is, of course, a sum beyond the means of the rank and file, but there is quite a large proportion of patients able to defray the full cost of the services rendered to them by their nurses, either in their own homes or in private institutions. For the rest we must look to the extension of the paying bed system, under which persons of moderate means may procure attendance without committing the crime of devitalising their nurses and cutting short their period of usefulness by using up the energies of ten years in as many months.

As the nursing services become better organised we shall realise how unspeakably foolish it is to perfect a priceless human instrument only to blunt and ruin it by setting it to perform impossibilities. We have experimented quite long enough in the dangerous direction of ascertaining the limits of a nurse's endurance. Let us now begin to put the lessons we have learned into practice.

